# Symptoms and quality of life before, during, and after a SARS-CoV-2 PCR positive or negative test: data from Lifelines

**DOI:** 10.1038/s41598-023-38223-5

**Published:** 2023-07-20

**Authors:** Yvonne M. J. Goërtz, Martijn A. Spruit, Maarten Van Herck, Nicole Dukers-Muijrers, Lifelines Corona Research Initiative, H. Marike Boezen, Jochen O. Mierau, H. Lude Franke, Jackie Dekens, Patrick Deelen, Pauline Lanting, Judith M. Vonk, Ilja Nolte, Anil P. S. Ori, Annique Claringbould, Floranne Boulogne, Marjolein X. L. Dijkema, Henry H. Wiersma, Robert Warmerdam, Soesma A. Jankipersadsing, Irene van Blokland, Geertruida H. de Bock, Cisca Wijmenga, Carla J. H. van der Kallen, Chris Burtin, Daisy J. A. Janssen

**Affiliations:** 1 Department of Research and Development, Ciro, Hornerheide 1, 6085 NM Horn, The Netherlands; 2 NUTRIM School of Nutrition and Translational Research in Metabolism, Maastricht University, Maastricht, The Netherlands; 3 Department of Respiratory Medicine, Maastricht University Medical Centre (MUMC+), Maastricht, The Netherlands; 4 REVAL – Rehabilitation Research Center, BIOMED – Biomedical Research Institute, Faculty of Rehabilitation Sciences, Hasselt University, Diepenbeek, Belgium; 5 CAPHRI Care and Public Health Research Institute, Maastricht University, Maastricht, The Netherlands; 6 Department of Sexual Health, Infectious Diseases and Environmental Health, Public Health Service South Limburg, Heerlen, The Netherlands; 7 Department of Health Promotion, Maastricht University, Maastricht, The Netherlands; 8 Department of Internal Medicine, Maastricht University Medical Centre (MUMC+), Maastricht, The Netherlands; 9 CARIM School for Cardiovascular Diseases, Maastricht University, Maastricht, The Netherlands; 10 Department of Health Services Research, Care and Public Health Research Institute, Faculty of Health, Medicine and Life Sciences, Maastricht University, Maastricht, The Netherlands; 11 Department of Epidemiology, University of Groningen, University Medical Center Groningen, Groningen, The Netherlands; 12 Department of Economics, Econometrics & Finance, Faculty of Economics and Business, University of Groningen, Groningen, The Netherlands; 13 Lifelines Cohort Study and Biobank, Groningen, The Netherlands; 14 Department of Genetics, University of Groningen, University Medical Center Groningen, Groningen, The Netherlands; 15 Department of Psychiatry, University of Groningen, University Medical Center Groningen, Groningen, The Netherlands; 16 Center of Development and Innovation, University of Groningen, University Medical Center Groningen, Groningen, The Netherlands; 17 Department of Cardiology, University of Groningen, University Medical Center Groningen, Groningen, The Netherlands; 18 Team Strategy & External Relations, University of Groningen, University Medical Center Groningen, Groningen, The Netherlands

**Keywords:** Fatigue, Infectious diseases, Viral infection

## Abstract

This study evaluates to what extent symptoms are present before, during, and after a positive SARS-CoV-2 polymerase chain reaction (PCR) test, and to evaluate how the symptom burden and quality of Life (QoL) compares to those with a negative PCR test. Participants from the Dutch Lifelines COVID-19 Cohort Study filled-out as of March 2020 weekly, later bi-weekly and monthly, questions about demographics, COVID-19 diagnosis and severity, QoL, and symptoms. The study population included those with one positive or negative PCR test who filled out two questionnaires before and after the test, resulting in 996 SARS-CoV-2 PCR positive and 3978 negative participants. Nearly all symptoms were more often reported after a positive test *versus* the period before the test (p < 0.05), except fever. A higher symptom prevalence after *versus* before a test was also found for nearly all symptoms in negatives (p < 0.05). Before the test, symptoms were already partly present and reporting of nearly all symptoms before did not differ between positives and negatives (p > 0.05). QoL decreased around the test for positives and negatives, with a larger deterioration for positives. Not all symptoms after a positive SARS-CoV-2 PCR test might be attributable to the infection and symptoms were also common in negatives.

## Introduction

Severe acute respiratory syndrome coronavirus 2 (SARS-CoV-2), the virus that causes coronavirus disease 2019 (COVID-19), manifests during the acute infection as heterogeneous with a wide spectrum of respiratory and non-respiratory related symptoms^1,2^. The severity of these symptoms can range from mild to severe or even fatal^3,4^. Fortunately, most patients fully recover. Nevertheless, it is becoming increasingly evident that a substantial proportion of people continues to experience symptoms for several weeks or months following the initial infection^5–8^. This is often referred to as long-COVID or post-COVID-19 condition, amongst others^9,10^. Commonly reported symptoms include fatigue, dyspnoea, cough, headache, brain fog, and this list is still growing^5–8^. These long-lasting symptoms negatively impact everyday functioning and quality of life (QoL)^7,11,12^.

The latest clinical case definition of the post COVID-19 condition proposed by the World Health Organization (WHO, dated 6th of October 2021) touches upon the nature of these persisting symptoms by stating that “…*symptoms may be new onset following initial recovery from an acute COVID-19 episode or persist from the initial illness. Symptoms may also fluctuate or relapse over time”*^10^*.* Nevertheless, to date it remains to be determined whether symptoms were already present prior to a SARS-CoV-2 infection, as prospective data are scarce^13^. In addition, few studies have investigated how the symptom burden differs between subjects with a positive and negative test before, during, and after a SARS-CoV-2 polymerase chain reaction (PCR) test^14,15^. Some of the persisting symptoms, such as headache, are not disease-specific, are common in the general population and can be aggravated due to another viral infection or underlying chronic disease, ageing, and/or the effect of the pandemic itself (e.g. lockdowns)^16^. We aimed to study to what extent symptoms are present before, during, and after a positive SARS-CoV-2 polymerase chain reaction (PCR) test, and to evaluate how the symptom burden and QoL compares to those with a negative PCR test.

## Results

167,729 people are enrolled in the Lifelines Cohort Study, of whom 139,375 were invited as of March 2020 for the first questionnaire of the Lifelines COVID-19 sub-study. Including only the eligible respondents for analyses, see Online Supplement Fig. 1, the study population comprised 996 SARS-CoV-2 PCR positive and 3978 negative participants. Participants with a SARS-CoV-2 PCR positive test were on average three years younger, slightly more often female, and had a higher BMI, compared to SARS-CoV-2 PCR negative participants. Education level was comparable between the SARS-CoV-2 positive and negative participants. Of the SARS-CoV-2 PCR positive tested participants 29 were hospitalized (3%). Of these, 23 participants used antibiotics, 25 participants used supplemental oxygen, and 11 participants were admitted to the ICU. Further details regarding demographical characteristics can be found in Table 1.

**Table 1:** Baseline characteristics stratified for a SARS-CoV-2 PCR positive (n = 996) or negative (n = 3978) test.

	N	SARS-CoV-2 PCR positive n (%)	N	SARS-CoV-2 PCR negative n (%)
Women, n (%)	996	660 (66·3)	3978	2619 (65·8)
Age (years), mean ± SD (min–max)	996	53·2 ± 11·4 (18–84)	3978	56·4 ± 11·6 (18–88)
BMI (kg/m^2^), mean ± SD	915	26·4 ± 4·5	3725	25·9 ± 4·1
Educational level, n (%)	959		3852	
Low		119 (12·4)		399 (10·4)
Medium		519 (54·1)		2035 (52·8)
High		321 (33·5)		1418 (36·8)
Health status at baseline, n (%)	784		3164	
Poor/mediocre		45 (5·7)		173 (5·5)
Good		400 (51·0)		1693 (53·5)
Very good		247 (31·5)		974 (30·8)
Excellent		92 (11·7)		324 (10·2)
Number of questionnaires between test and previous questionnaire, median (min–max)	996	2 (2–15)	3978	2 (2–15)
Number of questionnaires between test and following questionnaire, median (min–max)	996	1 (1–9)	3978	1 (1–10)

### Symptom prevalence

**Figure 1:**
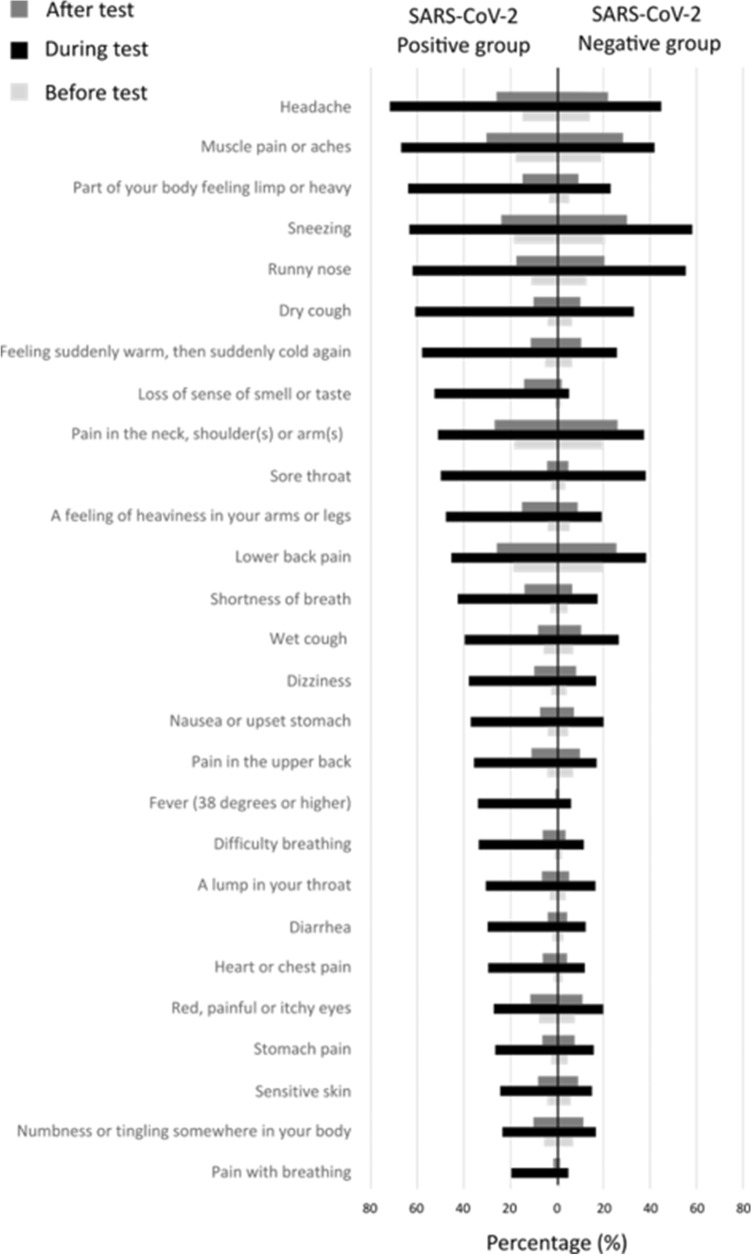
Prevalence of reported symptoms before, during, and after a SARS-CoV-2 PCR positive (n = 996) and negative (n = 3978) test.

In the weeks or months before the test, the symptom prevalence did not significantly differ between SARS-CoV-2 PCR positive and negative participants (p > 0.05), except for fever and pain in the upper back (p < 0.05) (Fig. 1). During the test, prevalence rates were significantly higher for all symptoms in positive compared to negative tested participants (for all: p < 0.05). In the weeks or months after the test, symptoms with significantly higher prevalence in positives *versus* negatives were, loss of sense of smell or taste, shortness of breath, a feeling of heaviness in your arms or legs, part of your body feeling limp or heavy, headache, difficulty breathing, heart or chest pain, dizziness, a lump in your throat, and pain with breathing (all p < 0.05, Fig. 1, see Online Supplement Table 1 for the symptom severity before, during, and after a SARS-CoV-2 positive and negative test).

### Change in symptom prevalence

Online Supplement Fig. 2 visually depicts the change in prevalence of symptoms over the time-periods by test-result. Before the test, symptoms were already partly present. Nearly all symptoms were significantly more often reported after a positive test compared to the period before the test (p < 0.05), except for fever (p > 0.05). The increase in symptom prevalence after compared to before a positive test ranges from 1% for sore throat (3% before *versus* 4% after the test, p < 0.05) to 13% for loss of sense of smell or taste (1% before *versus* 14% after, p < 0.05) and muscle pain or aches (18% before *versus* 31% after, p < 0·05). A higher symptom prevalence after compared to before a test was also found for nearly all symptoms in negative tested participants (p < 0.05), besides loss of sense of smell or taste (1% before *versus* 1% after, p > 0.05) and fever (< 1% before *versus* < 1% after, p < 0.05) which remained equal before and after a negative SARS-CoV-2 PCR test.

### Symptom burden

**Figure 2:**
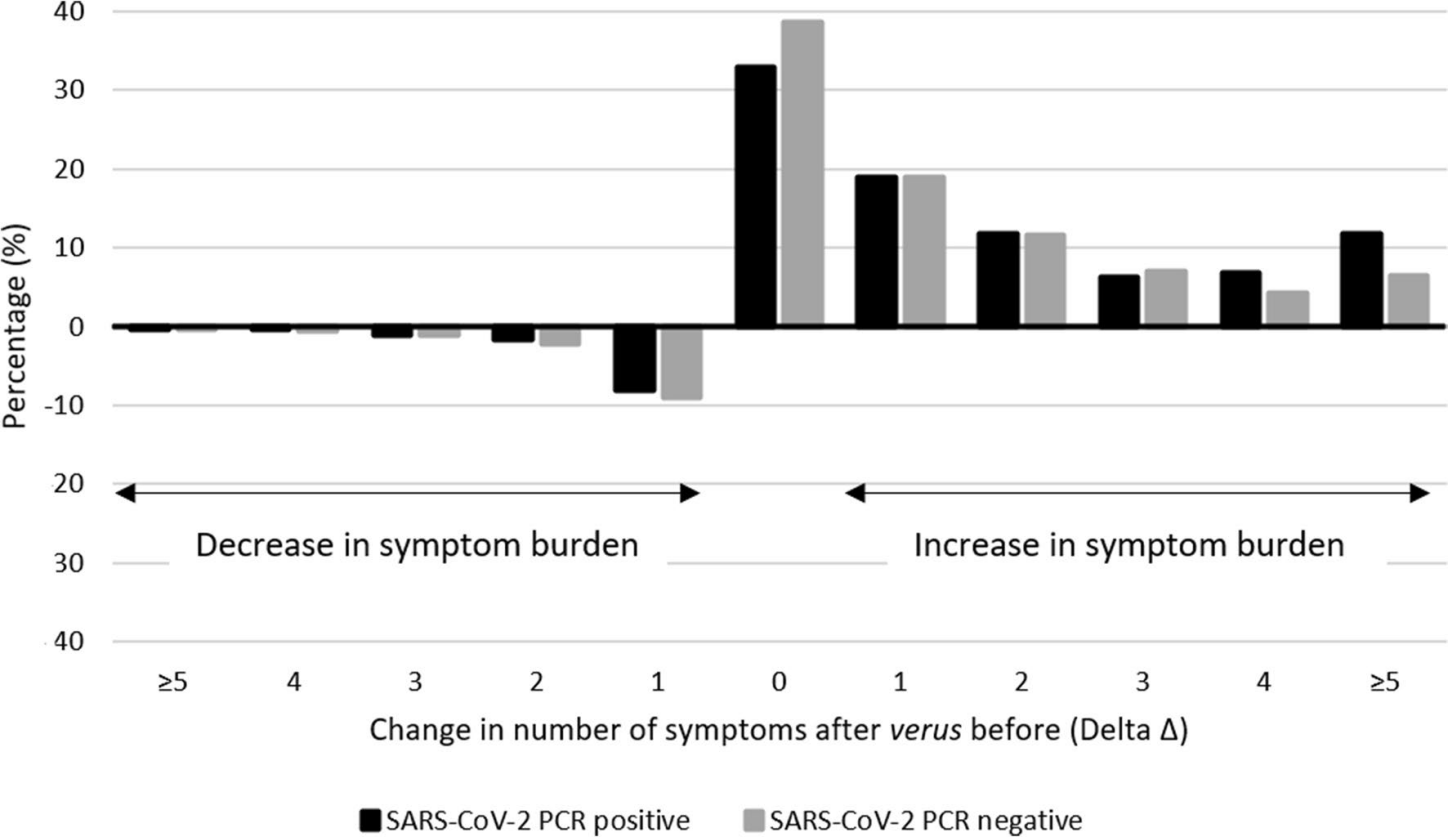
Change in total number of symptoms after *versus* before a SARS-CoV-2 PCR positive (n = 996) and negative (n = 3978) test.

Participants with a positive SARS-CoV-2 PCR test reported a median number of 1[0–2] symptom before, 12[7–16] symptoms during, and 2[0–5] symptoms after the test (*p* < 0·05). Participants with a negative SARS-CoV-2 PCR test experienced 1[0–2] symptom before, 5[3–9] symptoms during, and 2[0–4] symptoms after the test (*p* < 0.05). In the weeks to months following the testing, the total number of symptoms remained equal or decreased in 44% of positive and 52% of negative tested participants compared to pre-test levels (Fig. 2).

### Fatigue

**Figure 3:**
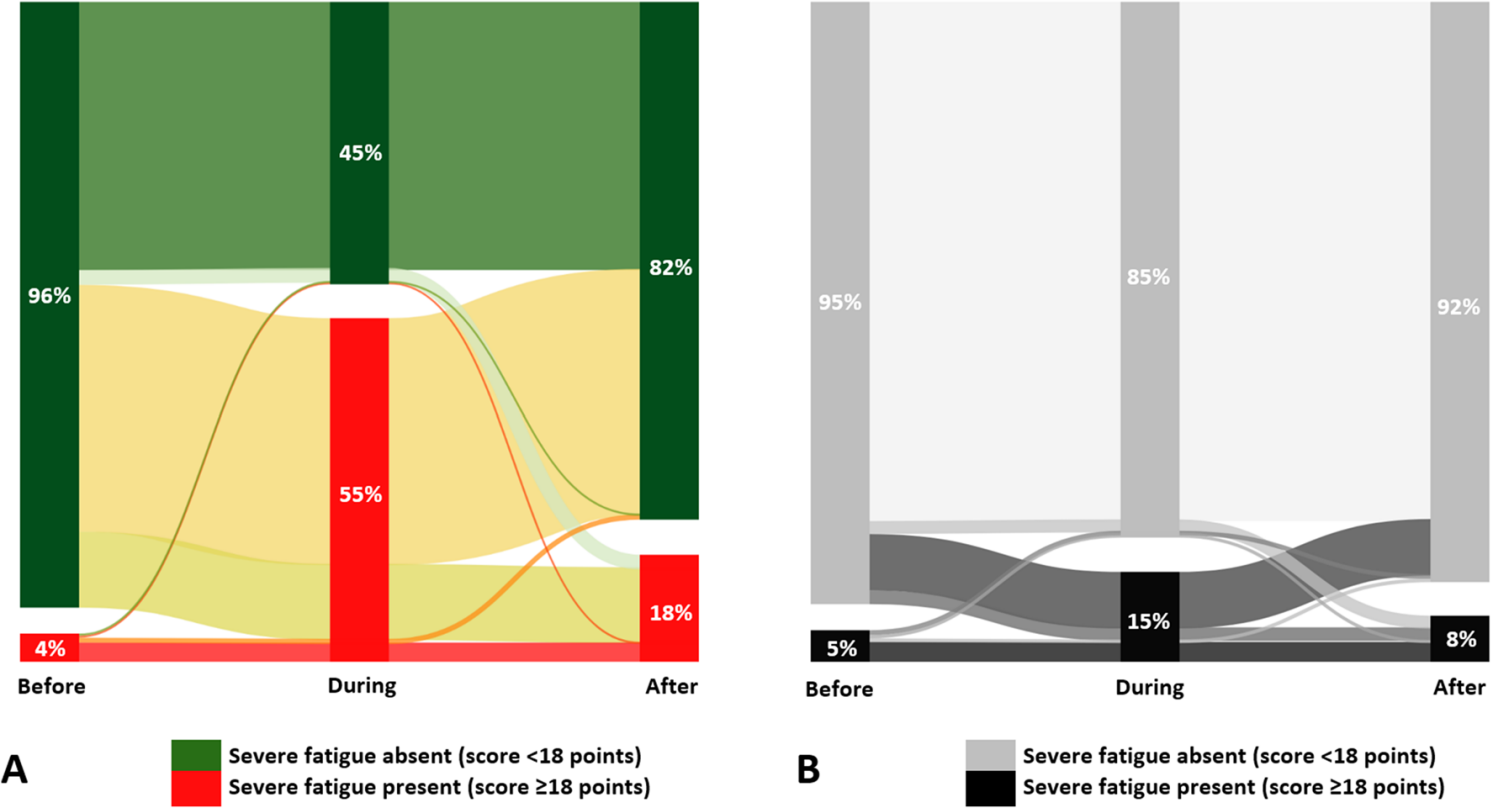
Prevalence and change of the symptom fatigue before, during, and after a SARS-CoV-2 PCR positive (**A**) and negative (**B**) test. The width of the lines is proportional to the flow rate. Severe fatigue is indicated by a SFQ score of ≥ 18 points.

The prevalence of severe fatigue was significantly increased after a positive test compared to the period before (4% *versus* 18% severe fatigue; p < 0.05). A higher prevalence of severe fatigue after compared to before the test was also found in negative tested participants, though to a lesser degree (5% *versus* 8%; *p* < 0.05) (Fig. 3).

### Quality of life

**Figure 4:**
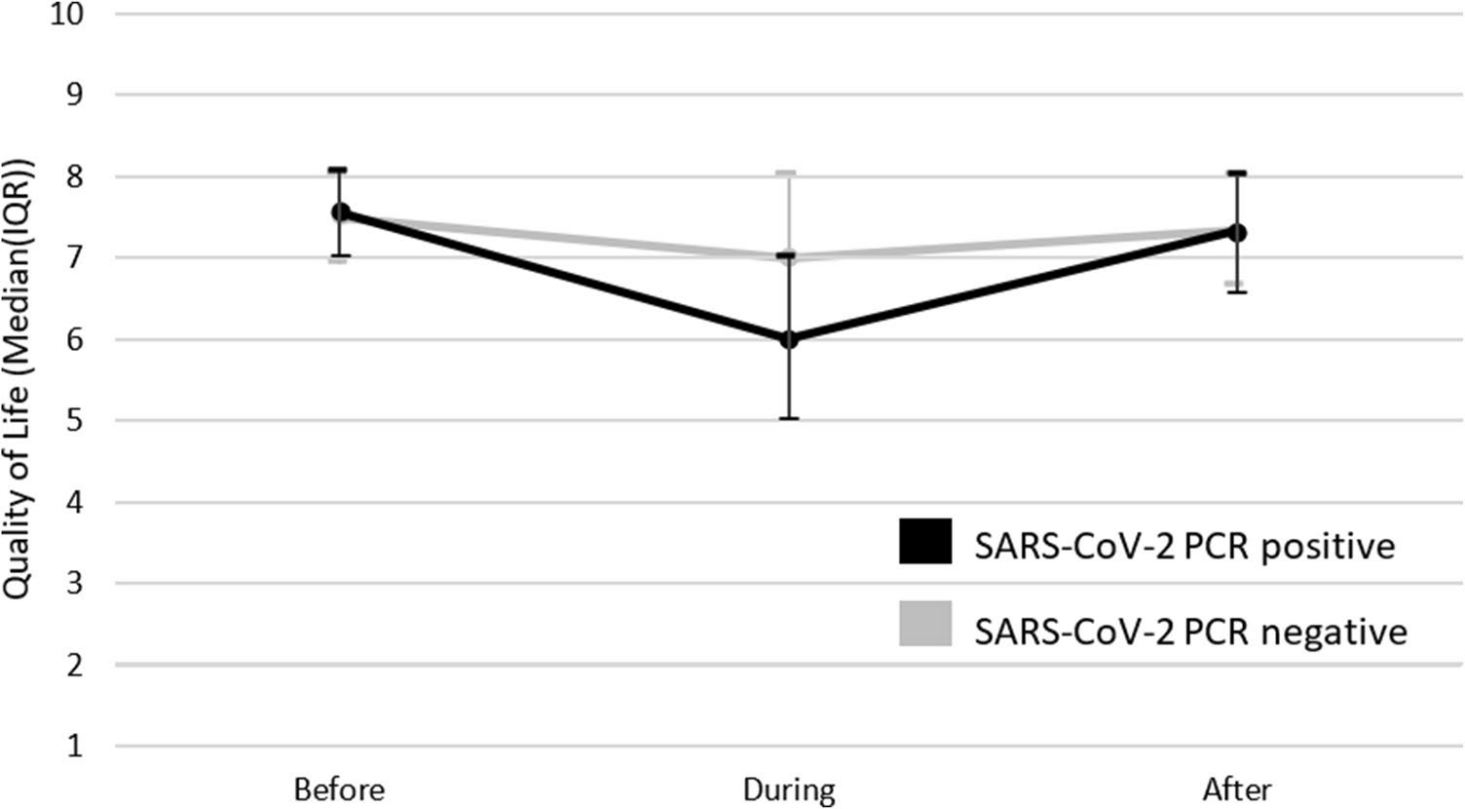
Quality of Life before, during, and after a SARS-CoV-2 PCR positive (n = 996) and negative (n = 3978) test. *IQR*  inter quartile range, SARS-CoV-2-PCR  severe acute respiratory syndrome coronavirus 2 polymerase chain reaction. Scale: 1 = terrible, 10 = excellent.

On average the experienced QoL was 8[7-8] points before, 6[5–7] points during, and 7[7-8] points after a positive SARS-CoV-2-PCR test (*p* < 0.05). For negative tested persons the QoL was lower during the test (though higher than in positives, i.e. before: 8[7-8], during: 7[6–8], after: 7[7-8], p < 0.05) (Fig. 4).

## Discussion

The study design of the Lifelines COVID-19 Cohort allows us to get a first detailed insight in the symptom burden and QoL before, during, and after a positive and negative SARS-CoV-2 PCR test. In the weeks or months following a SARS-CoV-2 infection, nearly all symptoms were more frequently present compared to before. A higher symptom prevalence after compared to before a test was also found for nearly all symptoms in negative tested participants. Before the test, symptoms were already partly present and reporting of nearly all symptoms before did not differ between positives and negatives. QoL decreased around the SARS-CoV-2 PCR test for both positive and negative tested participants, with a larger deterioration for positives.

The current study re-confirms that SARS-CoV-2 presents itself as a heterogeneous disease^1,2^. Fortunately, many positive tested participants fully recover. Nevertheless, a substantial proportion of participants continues to experience symptoms in the weeks or months after a positive SARS-CoV-2 PCR test. This is in accordance with previous studies performed in patients with mild to severe COVID-19^5–8^. Thus far, it was unknown whether and to what extent positive tested participants were free of symptoms before the actual infection, as data about the pre COVID-19 status in longitudinal studies were scarce. The current study showed that symptoms were already partly present before the infection. This implies that these persisting symptoms may not solely be attributed to the SARS-CoV-2 infection. This is in accordance with Wu and colleagues, who found that 44% of a sample from the U.S. community experienced at least one symptom already before the infection^13^. Then again, a part of the ongoing symptoms could not be explained by the pre-infection status and are, therefore, most probably new-onset or persisting symptoms following a SARS-CoV-2 infection.

Another difficulty when characterizing Long-COVID is the lack of data from participants with a negative SARS-CoV-2 test, as many symptoms are non-specific, common in the general population, and may be related to another infection, an underlying chronic disease, ageing, or changes in behaviour due to the pandemic^16^. Indeed, an increase in symptom burden was also found for participants after a negative SARS-CoV-2 PCR test compared to before, though not for the symptoms loss of sense of smell or taste and fever which remained equally prevalent. This is in accordance with Søraas and colleagues, who studied the persistence of symptoms 3 to 8 months after a SARS-CoV-2 positive or negative test and found that especially the symptom loss of sense of smell or taste was significantly more frequently reported by positive tested persons compared to negative 132 days after testing^15^. In addition, a French population-based cohort, comparing groups according to both European Center for Diseases Control (ECDC) criteria for COVID-19 (ECDC + or ECDC–) and serological SARS-CoV-2 tests results (sero + or sero–), found that individuals in all groups had a similar risk of having at least one symptom lasting more than 2 months^17^. Of note, these results should be interpreted with caution as some individuals do not show sero-conversion or because sero-reconversion occurs over time, leading to serological misclassification. Nevertheless, all these findings together suggest that persistent symptoms are also common in the general population and may, therefore, not all be directly related to a SARS-CoV-2 infection.

With regards to QoL, it was expected that participants with a positive SARS-CoV-2 PCR test would experience lower levels of QoL after the infection, compared to negative tested participants. The current findings, however, indicated that the QoL levels for both positive and negative tested participants nearly returned to pre-test values on group level. This while Huang et al. showed that community-dwelling COVID-19 survivors still had lower health status one year after the infection than non-COVID-19 controls^14^. Nevertheless, the included participants in the study from Huang and colleagues were all previously hospitalized patients, whereas the proportion of hospitalized participants in the current study was small, which could explain the difference in experienced health status in the weeks or months following the infection.

Some limitations have to be considered when interpreting the current findings. First, SARS-CoV-2 diagnosis and severity is based on self-report without the use of medical records. In addition, the precise date of the COVID-19 diagnosis was unknown. Therefore, the precise duration of the symptoms post-COVID-19 was also unknown. Second, the results cannot be generalized to all people with COVID-19, as adolescents (< 18 years of age) were not included in the current analyses. Moreover, cases with severe SARS-CoV-2 were less likely to participate in the current study. Hence, the small proportion of hospitalized participants. Third, the participants with a negative SARS-CoV-2 PCR test should not be considered as healthy controls, since these participants were eligible for PCR testing. Nevertheless, the reason for testing (e.g. testing due to COVID-19 like symptoms, contact with persons with COVID-19 or travels) was unknown. In addition, SARS-CoV-2 serology tests for the retrospective diagnosis of COVID-19 were not performed in the current study and, therefore, an undetected infection during the first months of the COVID-19 pandemic cannot be completely ruled out. Fourth, only participants with one positive or negative SARS-CoV-2 PCR test were taken into account. Hence, results cannot be generalized to people with multiple periods with symptoms that could be attributed to infections. Fifth, data concerning pre-existing comorbidities are lacking and the effect of pre-existing comorbidities on the symptom burden could not be determined. Sixth, BMI was assessed during the first visit of the general Lifelines Cohort Study (e.g. recruitment period 2006–2013). Therefore, the effect of a change in BMI on symptom severity (i.e. back pain, shortness of breath) could not be evaluated. Seventh, no information was available on whether the participants received a vaccination during the course of the study. Then again, the protective effect of vaccinations against Long-COVID is unknown yet^18,19^. Eight, the onset of new COVID-19 variants was not considered. Nevertheless, the current data are from approximately September 2020 to July 2021, while Omicron presented itself in Europe in late 2021 and early 2022. At last, the list of symptoms might not be all-inclusive, as people with a SARS-CoV-2 infection may experience up to 203 different symptoms, including brain fog, concentration problems, and fluctuating symptoms like post-exertional malaise^7,20^.

## Conclusions

This study contributes to the rapidly developing knowledge on COVID-19 and its long-term consequences. The findings indicate that not all symptoms after a positive SARS-CoV-2 test might be attributable to the infection and symptoms were also common in participants with a negative SARS-CoV-2 PCR test. Nonetheless, a part of the ongoing symptoms could not be explained by the pre-infection status. These new-onset or persisting symptoms after a SARS-CoV-2 infection are a major public health concern and warrant attention.

## Methods

### Study design and participants

The current study used data from the Lifelines COVID-19 Cohort Study, an add-on study to the Lifelines Cohort Study^21,22^. The Lifelines Cohort Study is an ongoing multi-disciplinary prospective population-based cohort study examining the health and health-related behaviours of 167,729 persons living in the North of The Netherlands in a unique three-generation design. It employs a broad range of investigative procedures in assessing the biomedical, socio-demographic, behavioural, physical and psychological factors which contribute to the health and disease of the general population, with a special focus on multi-morbidity and complex genetics. The participants of the Lifelines Cohort Study were recruited between 2006 and 2013, through general practitioners and self-enrollment. Participants who were unable to understand the Dutch language, were not able to fill in questionnaires, not able to visit the general practitioner, had severe mental illness (i.e. not fully capable to make rational decisions), or who had limited life expectancy (< 5 years) due to severe illness were not considered eligible^21–23^.

For the add-on Lifelines COVID-19 Cohort Study digital questionnaires were sent out to all adult participants with a known e-mail address of the Lifelines Cohort Study. At the beginning questionnaires were sent out on a weekly basis starting March 2020, later bi-weekly starting June 2020, and monthly starting August 2020. In the current study data from 24 questionnaires were used, collected over a period from approximately March 2020 through July 2021. A description of the cohort and study specifications has been published previously^24^.

Of note, the current analyses only take into account participants who reported one positive or negative SARS-CoV-2 PCR test as of COVID-19 Questionnaire 13 which was sent out starting September 2020 during the second wave of the pandemic when PCR tests were more readily available in The Netherlands. In addition, only participants who filled out at least two questionnaires before and after a positive or negative SARS-CoV-2 PCR test were included in the population of analyses.

The Lifelines Cohort Study, including the Lifelines COVID-19 Cohort Study, was conducted according to the principles of the Declaration of Helsinki and approved by the Medical Ethics Committee of the University Medical Center Groningen, the Netherlands (number 2007/152). No additional ethical approval is needed to request data collected within the regular protocol of Lifelines. All participants signed an informed consent.

### Measures

#### Demographic characteristics

Sex, age, educational level (low, medium, and high^25^), and self-reported health status (poor, mediocre, good, very good, and excellent) were assessed during the first COVID-19 questionnaire (COVID-19 Questionnaire 1). Body Mass Index (BMI) was assessed during the first visit of the general Lifelines Cohort Study by a research assistant using a standardized protocol.

#### COVID-19 related characteristics

Participants were asked to self-report whether they were tested for COVID-19 and if the result was positive or negative, since the last time they filled out a questionnaire. As of November 2020, a question was added to evaluate if the test was performed by the Municipal Public Health Service in The Netherlands. This was to ensure that only participants with a positive or negative SARS-CoV-2 PCR test, not self-administered test (antigen), were taken into account in the current analyses. Before November 2020 antigen tests were rarely available so most participants are likely to have had a PCR test. In case of a reported positive SARS-CoV-2 test, the severity of the infection was assessed (e.g. hospitalization, the use of antibiotics/supplemental oxygen during hospitalization, and intensive care unit (ICU) admission).

#### Self-reported symptoms

Self-report questions were used to evaluate the extent to which the participants experienced 28 symptoms (symptom severity) in the last 7 days using a five-point Likert-scale (1 = not at all; 2 = a little bit; 3 = somewhat; 4 = quite a lot; 5 = very much) (See Online Supplement for the list of symptoms). When questionnaires were sent out bi-weekly and monthly instead of weekly, the reference period of the questions was adapted to the last 14 and 28 days, respectively. A cut-off score of ≥ 2 points (‘a little bit’) was used to determine the prevalence of the symptom at the time of questionnaire administration.

Fatigue severity was measured using the Shortened Fatigue Questionnaire (SFQ). The SFQ consists of four items scored on a seven-point Likert scale (“I feel tired”, “I tire easily”, “I feel fit”, and “I feel physically exhausted”). The score ranges from 4 to 28 points, with higher scores indicating more severe fatigue. Severe fatigue is indicated by a SFQ score of ≥ 18 points^26^.

#### Quality of life

QoL was assessed with the following question “how would you rate your quality of life over the past 7, respectively 14 and 28, days”, using a 10 point-Likert scale (1 = terrible, 10 = excellent).

### Defining the symptom severity, -prevalence, -burden and QoL before, during, after a positive or negative test

To determine the symptom severity and QoL during a positive and negative SARS-CoV-2 PCR test, a so-called time-point zero was defined. This was the first administered questionnaire in which the respondent reported a positive or negative PCR test since the last time they filled in a questionnaire. To calculate the symptom severity and QoL in the weeks/months prior and after a positive or negative SARS-CoV-2 PCR test, the average was calculated of the available answers on the Likert-scales from COVID-19 Questionnaire 1 to 24. In these calculations, information on symptoms and QoL from the questionnaire that was filled out directly before the test was not taken into account to prevent that an emerging infection influenced the symptom burden and QoL in the pre-test period. Based on the symptom severity in the weeks/months before, during, and after a PCR test, the prevalence of each symptom was determined using the cut-off score of ≥ 2 points (‘a little bit’). The total number of symptoms present before, during, or after a PCR test is referred to as the symptom burden.

### Statistical analyses

Statistical analyses and visualization were conducted using SPSS (V.25.0 for Windows, Chicago, IL, USA) and SankeyMATIC (http://sankeymatic.com/build/). Descriptive statistics on group level were reported as mean and standard deviation, median and interquartile range, or frequency and percentage, as appropriate. Chi-square was conducted to compare symptoms and QoL by test-result (e.g. positive *versus* negative tested participants) for each time-period separately. One-way repeated measured ANOVA and McNemar test were used to determine if there were differences in symptoms and QoL between time-periods (before, during, and after a SARS-CoV-2 test) for positives and negatives separately. In case of significant differences between time-periods, a post-hoc analysis with Bonferroni adjustment was carried out. The level of significance was set at < 0.05.

### Supplementary Information

Supplementary Information.

## Data Availability

We are not permitted to share individual data from the Dutch Lifelines study. Information on applying for access to the Dutch Lifelines data is available at https://www.lifelines.nl/researcher/how-to-apply.

## References

[1] Docherty AB (2020). Features of 20 133 UK patients in hospital with covid-19 using the ISARIC WHO Clinical Characterisation Protocol: Prospective observational cohort study. BMJ.

[2] Guan WJ (2020). Clinical characteristics of coronavirus disease 2019 in China. N. Engl. J. Med..

[3] Verity R (2020). Estimates of the severity of coronavirus disease 2019: A model-based analysis. Lancet Infect. Dis..

[4] WHO (2020). Working Group on the Clinical Characterisation and Management of COVID-19 infection. A minimal common outcome measure set for COVID-19 clinical research. Lancet Infect. Dis..

[5] Carfì A, Bernabei R, Landi F (2020). Persistent symptoms in patients after acute COVID-19. JAMA.

[6] Vaes AW (2021). Recovery from COVID-19: A sprint or marathon? 6-month follow-up data from online long COVID-19 support group members. ERJ Open Res..

[7] Davis HE (2021). Characterizing long COVID in an international cohort: 7 months of symptoms and their impact. EClinicalMedicine.

[8] Goërtz YMJ (2020). Persistent symptoms 3 months after a SARS-CoV-2 infection: The post-COVID-19 syndrome?. ERJ Open Res..

[9] Callard F, Perego E (2021). How and why patients made long Covid. Soc. Sci. Med..

[10] WHO. *A clinical case definition of post COVID-19 condition by a Delphi consensus*. https://apps.who.int/iris/rest/bitstreams/1376291/retrieve (Accessed 6 October 2021). 10.1016/S1473-3099(21)00703-9 PMC8691845 34951953

[11] Meys R (2020). Generic and respiratory-specific quality of life in non-hospitalized patients with COVID-19. J. Clin. Med..

[12] Delbressine JM (2021). The impact of post-COVID-19 syndrome on self-reported physical activity. Int. J. Environ. Res. Public Health.

[13] Wu Q, Ailshire J, Crimmins E (2022). Long COVID and symptom trajectory in a representative sample of Americans. Sci. Rep..

[14] Huang L (2021). 1-year outcomes in hospital survivors with COVID-19: A longitudinal cohort study. Lancet.

[15] Søraas A (2021). Persisting symptoms three to eight months after non-hospitalized COVID-19, a prospective cohort study. PLoS One.

[16] Amin-Chowdhury Z, Ladhani SN (2021). Causation or confounding: Why controls are critical for characterizing long COVID. Nat. Med..

[17] Robineau O (2022). Persistent symptoms after the first wave of COVID-19 in relation to SARS-CoV-2 serology and experience of acute symptoms: A nested survey in a population-based cohort. Lancet Reg. Health Eur..

[18] Strain WD (2022). The impact of COVID vaccination on symptoms of long COVID: An international survey of people with lived experience of long COVID. Vaccines.

[19] Taquet M, Dercon Q, Harrison PJ (2022). Six-month sequelae of post-vaccination SARS-CoV-2 infection: A retrospective cohort study of 10,024 breakthrough infections. Brain Behav. Immun..

[20] Brown DA, O'Brien KK (2021). Conceptualising long COVID as an episodic health condition. BMJ Glob. Health.

[21] Stolk RP (2008). Universal risk factors for multifactorial diseases: LifeLines: A three-generation population-based study. Eur. J. Epidemiol..

[22] Scholtens S (2015). Cohort profile: LifeLines, a three-generation cohort study and biobank. Int. J. Epidemiol..

[23] Klijs B (2015). Representativeness of the LifeLines Cohort Study. PLoS One.

[24] Mc Intyre K (2021). Lifelines COVID-19 cohort: Investigating COVID-19 infection and its health and societal impacts in a Dutch population-based cohort. BMJ Open.

[25] International Standard Classification of Education ISCED. http://uis.unesco.org/sites/default/files/documents/international-standard-classification-of-education-isced-2011-en.pdf (Accessed 6 October 2011).

[26] Penson A (2020). Short fatigue questionnaire: Screening for severe fatigue. J. Psychosom. Res..

