# Author Correction: Symptoms and quality of life before, during, and after a SARS‑CoV‑2 PCR positive or negative test: data from Lifelines

**DOI:** 10.1038/s41598-024-58848-4

**Published:** 2024-04-10

**Authors:** Yvonne M. J. Goërtz, Martijn A. Spruit, Maarten Van Herck, Nicole Dukers-Muijrers, Lifelines Corona Research Initiative, H. Marike Boezen, Jochen O. Mierau, H. Lude Franke, Jackie Dekens, Patrick Deelen, Pauline Lanting, Judith M. Vonk, Ilja Nolte, Anil P. S. Ori, Annique Claringbould, Floranne Boulogne, Marjolein X. L. Dijkema, Henry H. Wiersma, Robert Warmerdam, Soesma A. Jankipersadsing, Irene van Blokland, Geertruida H. de Bock, Cisca Wijmenga, Carla J. H. van der Kallen, Chris Burtin, Daisy J. A. Janssen

**Affiliations:** 1 Department of Research and Development, Ciro, Hornerheide 1, 6085 NM Horn, The Netherlands; 2 NUTRIM School of Nutrition and Translational Research in Metabolism, Maastricht University, Maastricht, The Netherlands; 3 Department of Respiratory Medicine, Maastricht University Medical Centre (MUMC+), Maastricht, The Netherlands; 4 REVAL – Rehabilitation Research Center, BIOMED – Biomedical Research Institute, Faculty of Rehabilitation Sciences, Hasselt University, Diepenbeek, Belgium; 5 CAPHRI Care and Public Health Research Institute, Maastricht University, Maastricht, The Netherlands; 6 Department of Sexual Health, Infectious Diseases and Environmental Health, Public Health Service South Limburg, Heerlen, The Netherlands; 7 Department of Health Promotion, Maastricht University, Maastricht, The Netherlands; 8 Department of Internal Medicine, Maastricht University Medical Centre (MUMC+), Maastricht, The Netherlands; 9 CARIM School for Cardiovascular Diseases, Maastricht University, Maastricht, The Netherlands; 10 Department of Health Services Research, Care and Public Health Research Institute, Faculty of Health, Medicine and Life Sciences, Maastricht University, Maastricht, The Netherlands; 11 Department of Epidemiology, University of Groningen, University Medical Center Groningen, Groningen, The Netherlands; 12 Department of Economics, Econometrics & Finance, Faculty of Economics and Business, University of Groningen, Groningen, The Netherlands; 13 Lifelines Cohort Study and Biobank, Groningen, The Netherlands; 14 Department of Genetics, University of Groningen, University Medical Center Groningen, Groningen, The Netherlands; 15 Department of Psychiatry, University of Groningen, University Medical Center Groningen, Groningen, The Netherlands; 16 Center of Development and Innovation, University of Groningen, University Medical Center Groningen, Groningen, The Netherlands; 17 Department of Cardiology, University of Groningen, University Medical Center Groningen, Groningen, The Netherlands; 18 Team Strategy & External Relations, University of Groningen, University Medical Center Groningen, Groningen, The Netherlands

Correction to: *Scientific Reports* 10.1038/s41598-023-38223-5, published online 20 July 2023.

The original version of this Article contained an error in the Consortium list ‘Lifelines Corona Research Initiative’, in which Judith G. M. Rosmalen was incorrectly listed as an author. Subsequently, her name has been removed.

The original Article has been corrected.

