# Supplementary material for: Symptoms and quality of life before, during, and after a SARS-CoV-2 PCR positive or negative test: data from Lifelines
Source: Sci Rep. 2023 Jul 20;13:11713. doi: 10.1038/s41598-023-38223-5 (PMC10359407; doi:10.1038/s41598-023-38223-5)
Supplement: Supplementary file 1 — Supplementary Information. [file 41598_2023_38223_MOESM1_ESM.pdf]

# Online Supplement

**Symptoms and quality of life before, during and after a SARS-CoV-2 PCR positive or negative test: data from Lifelines**

Yvonne M.J. Goërtz, MSc[1,2,3], Prof. Martijn A. Spruit[1,2,3], Maarten Van Herck, MSc[1,2,3,4], Nicole Dukers-Muijrers, PhD[5,6,7], Lifelines Corona Research Initiative, Carla J.H. van der Kallen, PhD[8,9], Chris Burtin, PhD[4], Prof. Daisy J.A. Janssen, MD[1,10]

[1]Department of Research and Development, Ciro, Horn, the Netherlands

[2]NUTRIM School of Nutrition and Translational Research in Metabolism, Maastricht University, Maastricht, the Netherlands

[3]Department of Respiratory Medicine, Maastricht University Medical Centre (MUMC+), Maastricht, the Netherlands

[4]REVAL – Rehabilitation Research Center, BIOMED – Biomedical Research Institute, Faculty of Rehabilitation Sciences, Hasselt University, Diepenbeek, Belgium

[5]CAPHRI Care and Public Health Research Institute, Maastricht University, Maastricht, the Netherlands.

[6]Department of Sexual Health, Infectious Diseases and Environmental Health, Public Health Service South Limburg, Heerlen, the Netherlands

[7]Department of Medical Microbiology, Maastricht University Medical Centre (MUMC+), Maastricht, the Netherlands

[8]Department of Internal Medicine, Maastricht University Medical Centre (MUMC+), Maastricht, the Netherlands

[9]CARIM School for Cardiovascular Diseases, Maastricht University, Maastricht, the Netherlands

[10]Department of Health Services Research, Care and Public Health Research Institute, Faculty of Health, Medicine and Life Sciences, Maastricht University, Maastricht, the Netherlands.

**Online Supplement Methods**

**Self-reported symptoms**

Self-report questions were used to evaluate the extent to which the participants experienced symptoms (symptom severity) in the last 7, respectively 14 and 28 days, using a five-point Likert-scale (1=not at all; 2=a little bit; 3=somewhat; 4=quite a lot; 5=very much). **The following 27 symptoms were evaluated using a checklist: headache, dizziness, heart or chest pain, lower back pain, nausea or upset stomach, muscle pain or aches, difficulty breathing, feeling suddenly warm then suddenly cold again, numbness or tingling somewhere in your body, a lump in your throat, part of your body feeling limp or heavy, a feeling of heaviness in your arms or legs, sensitive skin, pain in the neck/shoulder(s)/arm(s), pain in the upper back, shortness of breath, pain with breathing, runny nose, sore throat, dry cough, wet cough, fever (38 degrees or higher), diarrhoea, stomach pain, loss of sense of smell or taste, red, painful or itchy eyes, and sneezing. Notably, as the general understanding of COVID-19 continued to evolve throughout the study, symptoms were added to the questionnaire. For this reason, the symptoms sensitive skin (COVQ 7-22), pain in the neck/shoulder(s)/arm(s) (COVQ 8-22), pain in the upper back (COVQ 8-22), diarrhoea (COVQ 3-22), stomach pain (COVQ 3-22) and sneezing (COVQ 2-22) were not available for every time point (e.g., COVQ 1-22).** Besides the symptom severity based on the five-point Likert scale, a cut-off score of ≥2 points ('a little bit') was used to determine the prevalence of the symptom at the time of questionnaire administration.

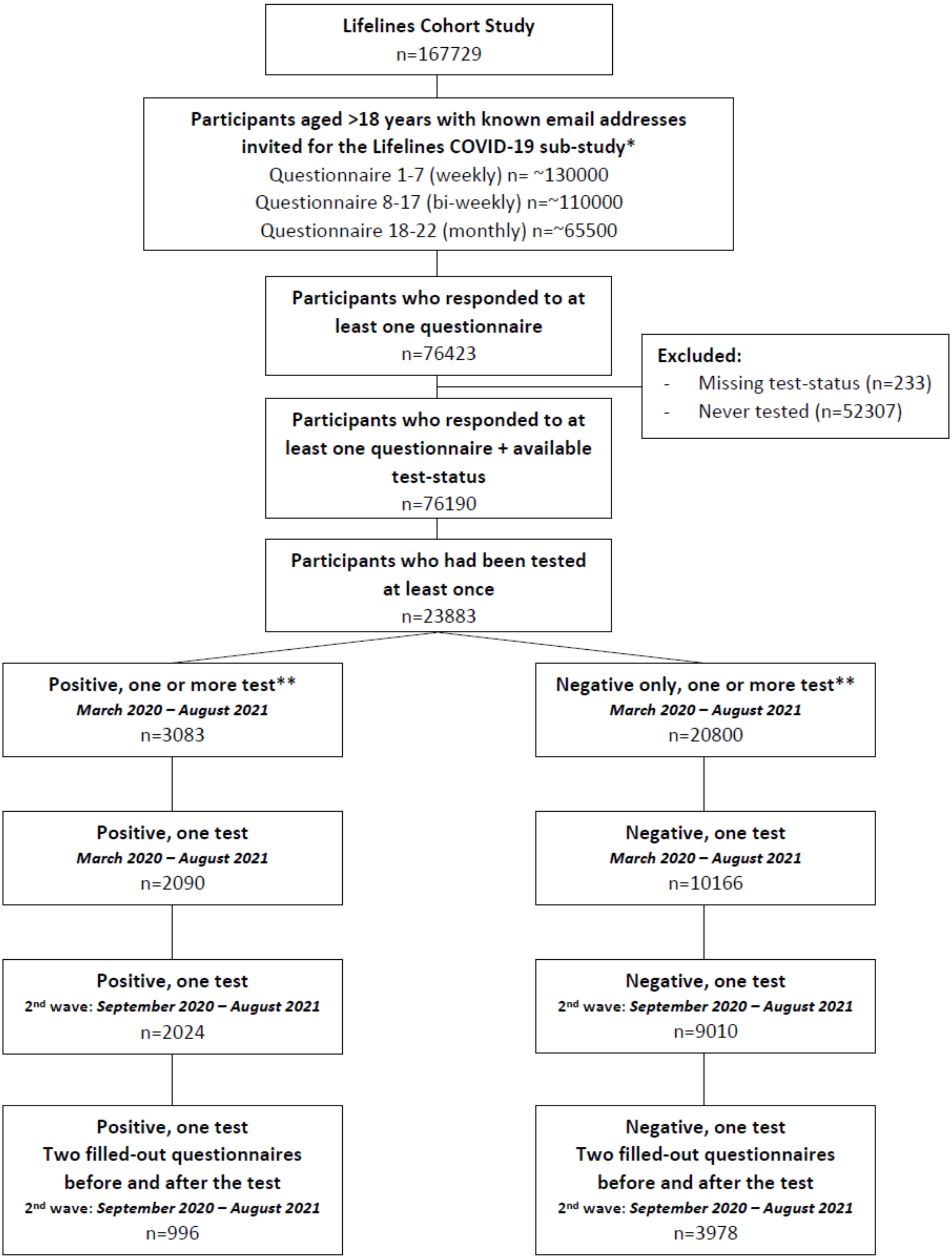


**Figure 1 Online Supplement: Flowchart of participants** **From COVID-19 Questionnaire 8 (starting May 2020) ~110000 participants were invited, from COVID-19 Questionnaire 18 (starting February 2021) ~65500 participants were invited.* **Self-reported SARS-CoV-2 PCR test result. Participants with a negative test-based COVID-19 infection never had a positive test during the complete study time.

**Table 1 Online Supplement: Symptom severity before, during, and after a SARS-CoV-2-PCR positive (n=996) and negative (n=3978) test.**

| | Positive tested people | | | | Negative tested people | | | |
|---|---|---|---|---|---|---|---|---|
| | n | Before | During | After | n | Before | During | After |
| | | mean±SD | mean±SD | mean±SD | | mean±SD | mean±SD | mean±SD |
| Headache | 996 | 1.46±0.49 | 2.32±1.19 | 1.56±0.66* | 3968 | 1.42±0.48 | 1.57±0.77 | 1.45±0.54* |
| Dizziness | 996 | 1.15±0.28 | 1.56±0.85 | 1.25±0.44* | 3968 | 1.17±0.31 | 1.19±0.48 | 1.19±0.37* |
| Heart or chest pain | 995 | 1.10±0.23 | 1.43±0.77 | 1.18±0.41* | 3967 | 1.10±0.24 | 1.14±0.42 | 1.10±0.28* |
| Lower back pain | 994 | 1.49±0.60 | 1.71±0.96 | 1.52±0.68* | 3971 | 1.49±0.61 | 1.51±0.77 | 1.52±0.66* |
| Nausea or upset stomach | 996 | 1.18±0.32 | 1.58±0.90 | 1.22±0.42* | 3968 | 1.18±0.33 | 1.25±0.58 | 1.19±0.38* |
| Muscle pain or aches | 994 | 1.49±0.58 | 2.26±1.20 | 1.63±0.71* | 3969 | 1.50±0.59 | 1.56±0.80 | 1.56±0.67* |
| Difficulty breathing | 994 | 1.08±0.21 | 1.49±0.82 | 1.18±0.40* | 3966 | 1.08±0.24 | 1.14±0.43 | 1.08±0.27* |
| Feeling suddenly warm, then suddenly cold again | 995 | 1.19±0.39 | 2.07±1.17 | 1.29±0.51* | 3967 | 1.20±0.40 | 1.33±0.65 | 1.24±0.48* |
| Numbness or tingling somewhere in your body | 995 | 1.19±0.37 | 1.33±0.69 | 1.24±0.47* | 3967 | 1.20±0.42 | 1.20±0.51 | 1.23±0.48* |
| A lump in your throat | 996 | 1.14±0.28 | 1.45±0.81 | 1.17±0.39* | 3966 | 1.13±0.31 | 1.20±0.50 | 1.12±0.32* |
| Part of your body feeling limp or heavy | 996 | 1.15±0.31 | 2.24±1.24 | 1.35±0.62* | 3965 | 1.18±0.38 | 1.31±0.65 | 1.22±0.44* |
| A feeling of heaviness in your arms or legs | 996 | 1.16±0.33 | 1.90±1.17 | 1.33±0.60* | 3968 | 1.18±0.37 | 1.25±0.59 | 1.21±0.44* |
| Sensitive skin | 939 | 1.14±0.32 | 1.41±0.86 | 1.19±0.39* | 3809 | 1.16±0.36 | 1.18±0.48 | 1.19±0.41* |
| Pain in the neck, shoulder(s) or arm(s) | 926 | 1.43±0.60 | 1.88±1.06 | 1.57±0.73* | 3731 | 1.44±0.61 | 1.50±0.78 | 1.52±0.68* |
| Pain in the upper back | 925 | 1.15±0.37 | 1.63±1.02 | 1.24±0.47* | 3726 | 1.17±0.40 | 1.22±0.57 | 1.21±0.47* |
| Shortness of breath | 996 | 1.13±0.29 | 1.66±0.95 | 1.31±0.56* | 3967 | 1.14±0.33 | 1.21±0.53 | 1.14±0.36* |
| Pain with breathing | 996 | 1.03±0.13 | 1.28±0.66 | 1.07±0.23* | 3968 | 1.02±0.13 | 1.05±0.30 | 1.02±0.14* |
| Runny nose | 996 | 1.37±0.43 | 1.89±0.90 | 1.38±0.45* | 3968 | 1.39±0.45 | 1.76±0.86 | 1.40±0.48* |
| Sore throat | 996 | 1.18±0.29 | 1.73±0.90 | 1.17±0.32* | 3969 | 1.17±0.28 | 1.51±0.78 | 1.15±0.28* |
| Dry cough | 996 | 1.21±0.32 | 1.98±1.05 | 1.24±0.42* | 3958 | 1.22±0.34 | 1.43±0.71 | 1.21±0.37* |
| Wet cough | 994 | 1.17±0.34 | 1.58±0.86 | 1.19±0.39* | 3965 | 1.18±0.34 | 1.34±0.66 | 1.18±0.38* |
| Fever (38 degrees or higher) | 995 | 1.01±0.09 | 1.65±1.09 | 1.04±0.15* | 3966 | 1.01±0.05 | 1.09±0.42 | 1.01±0.11* |
| Diarrhoea | 984 | 1.11±0.25 | 1.43±0.78 | 1.14±0.32* | 3941 | 1.11±0.25 | 1.14±0.44 | 1.12±0.29* |
| Stomach pain | 983 | 1.15±0.28 | 1.36±0.69 | 1.19±0.38* | 3948 | 1.16±0.33 | 1.20±0.52 | 1.18±0.38* |
| Loss of sense of smell or taste | 994 | 1.04±0.22 | 2.36±1.56 | 1.36±0.78* | 3967 | 1.04±0.23 | 1.07±0.34 | 1.04±0.25* |
| Red, painful or itchy eyes | 995 | 1.24±0.40 | 1.43±0.82 | 1.27±0.48* | 3968 | 1.24±0.42 | 1.24±0.56 | 1.23±0.44 |
| Sneezing | 989 | 1.51±0.46 | 1.83±0.79 | 1.47±0.49* | 3956 | 1.52±0.46 | 1.72±0.75 | 1.52±0.50* |

* $p$<0.05. n = number, SD = Standard Deviation.

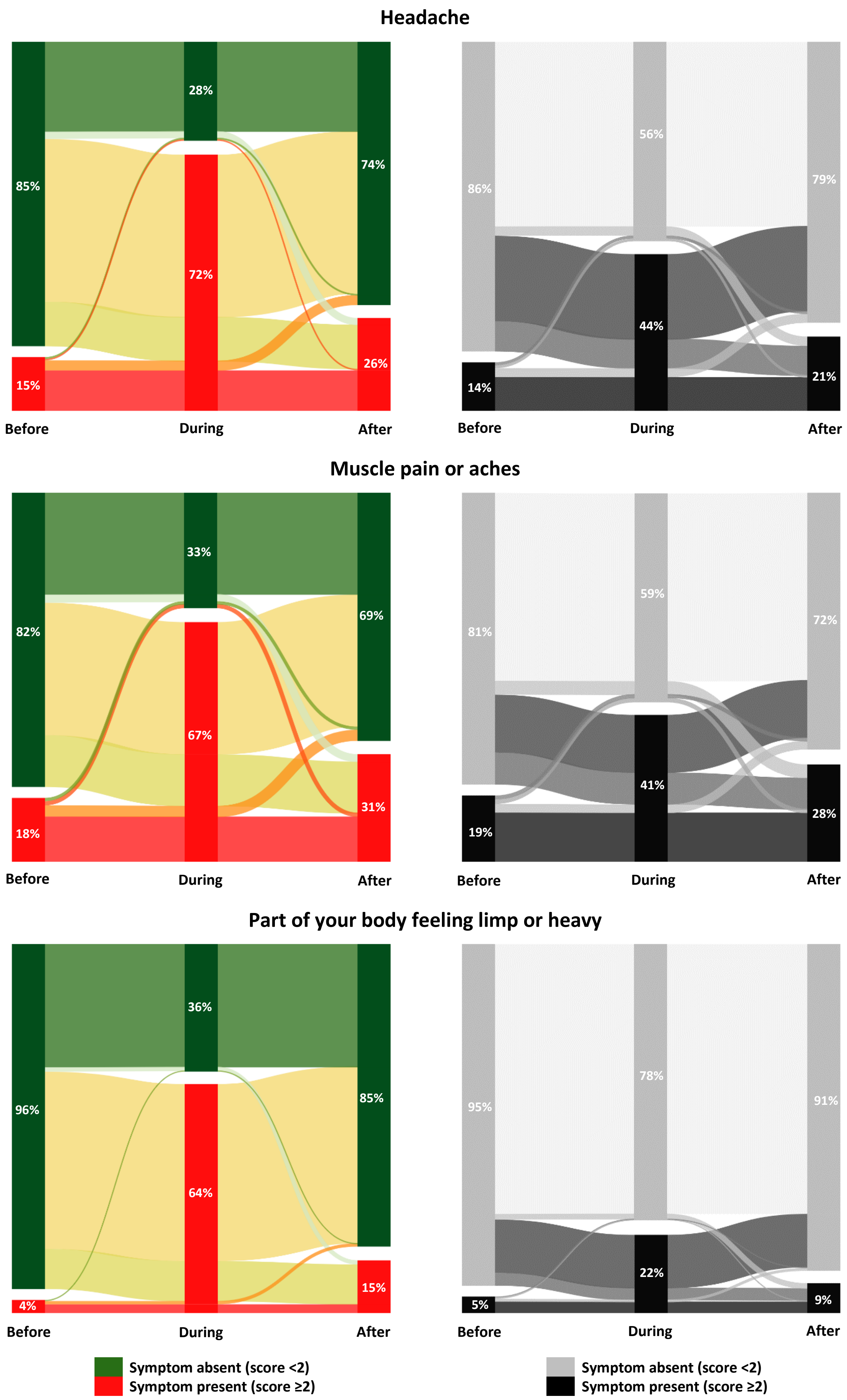
Headache
85%
15%
28%
72%
74%
26%
86%
14%
56%
44%
79%
21%
Before
During
After
Before
During
After
Muscle pain or aches
82%
18%
33%
67%
69%
31%
81%
19%
59%
41%
72%
28%
Before
During
After
Before
During
After
Part of your body feeling limp or heavy
96%
4%
36%
64%
85%
15%
95%
5%
78%
22%
91%
9%
Before
During
After
Before
During
After
Symptom absent (score <2)
Symptom present (score ≥2)
Symptom absent (score <2)
Symptom present (score ≥2)

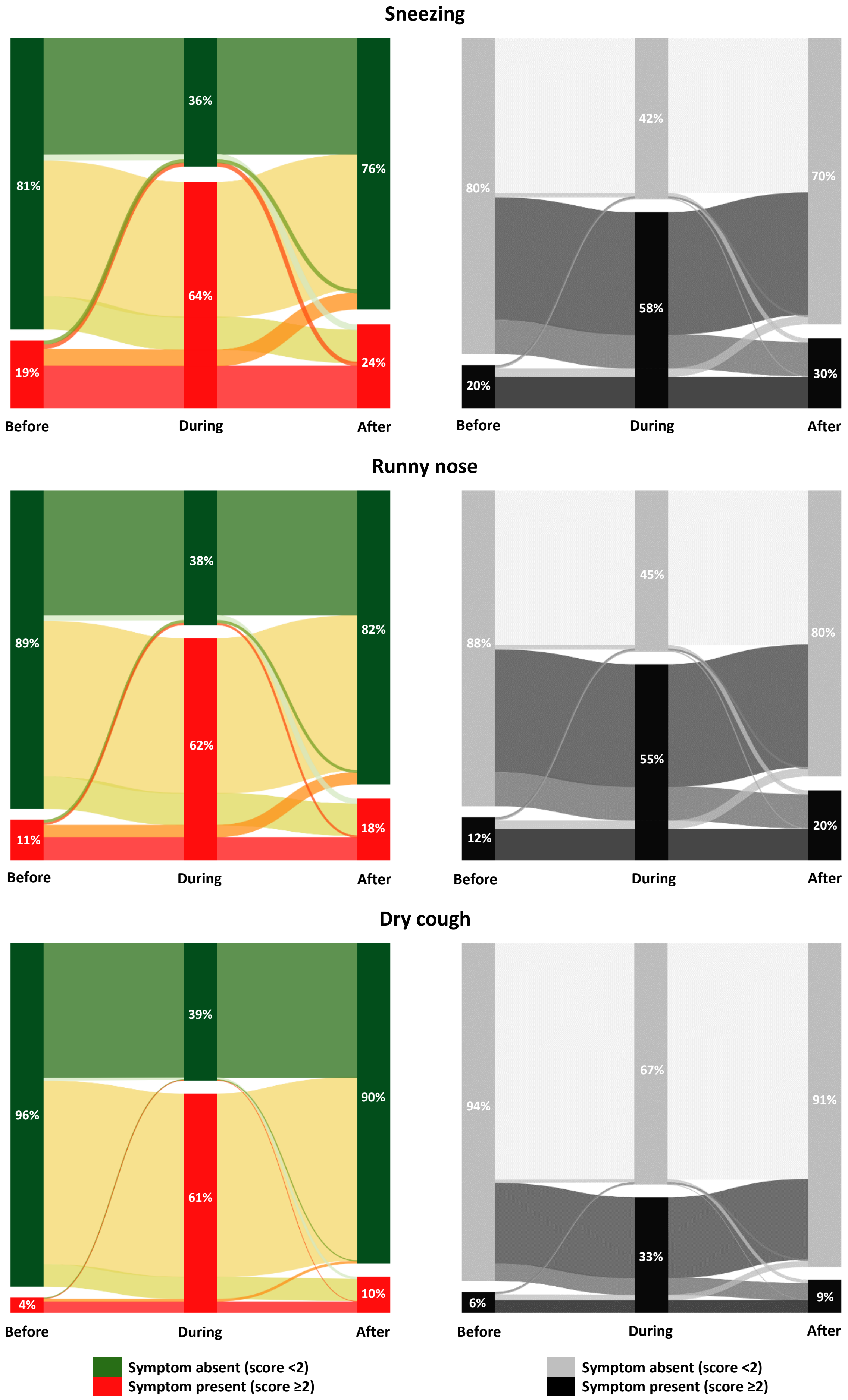

Sneezing
81%
19%
36%
64%
76%
24%
80%
20%
42%
58%
70%
30%
Before
During
After
Before
During
After
Runny nose
89%
11%
38%
62%
82%
18%
88%
12%
45%
55%
80%
20%
Before
During
After
Before
During
After
Dry cough
96%
4%
39%
61%
90%
10%
94%
6%
67%
33%
91%
9%
Before
During
After
Before
During
After
Symptom absent (score <2)
Symptom present (score ≥2)
Symptom absent (score <2)
Symptom present (score ≥2)

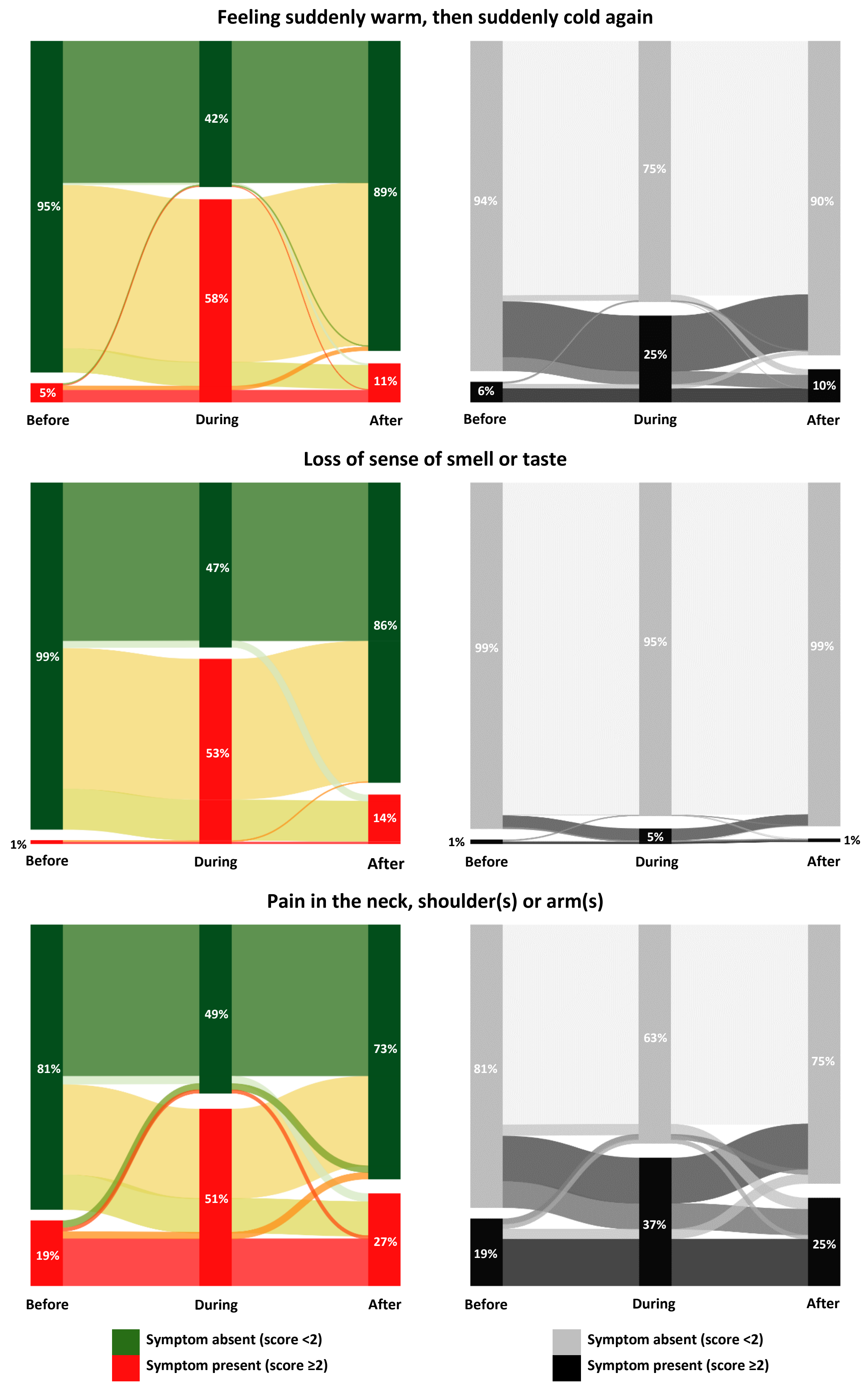

Feeling suddenly warm, then suddenly cold again
95%
5%
42%
58%
89%
11%
Before
During
After
94%
6%
75%
25%
90%
10%
Before
During
After
Loss of sense of smell or taste
99%
1%
47%
53%
86%
14%
Before
During
After
99%
1%
95%
5%
99%
1%
Before
During
After
Pain in the neck, shoulder(s) or arm(s)
81%
19%
49%
51%
73%
27%
Before
During
After
81%
19%
63%
37%
75%
25%
Before
During
After
Symptom absent (score <2)
Symptom present (score ≥2)
Symptom absent (score <2)
Symptom present (score ≥2)

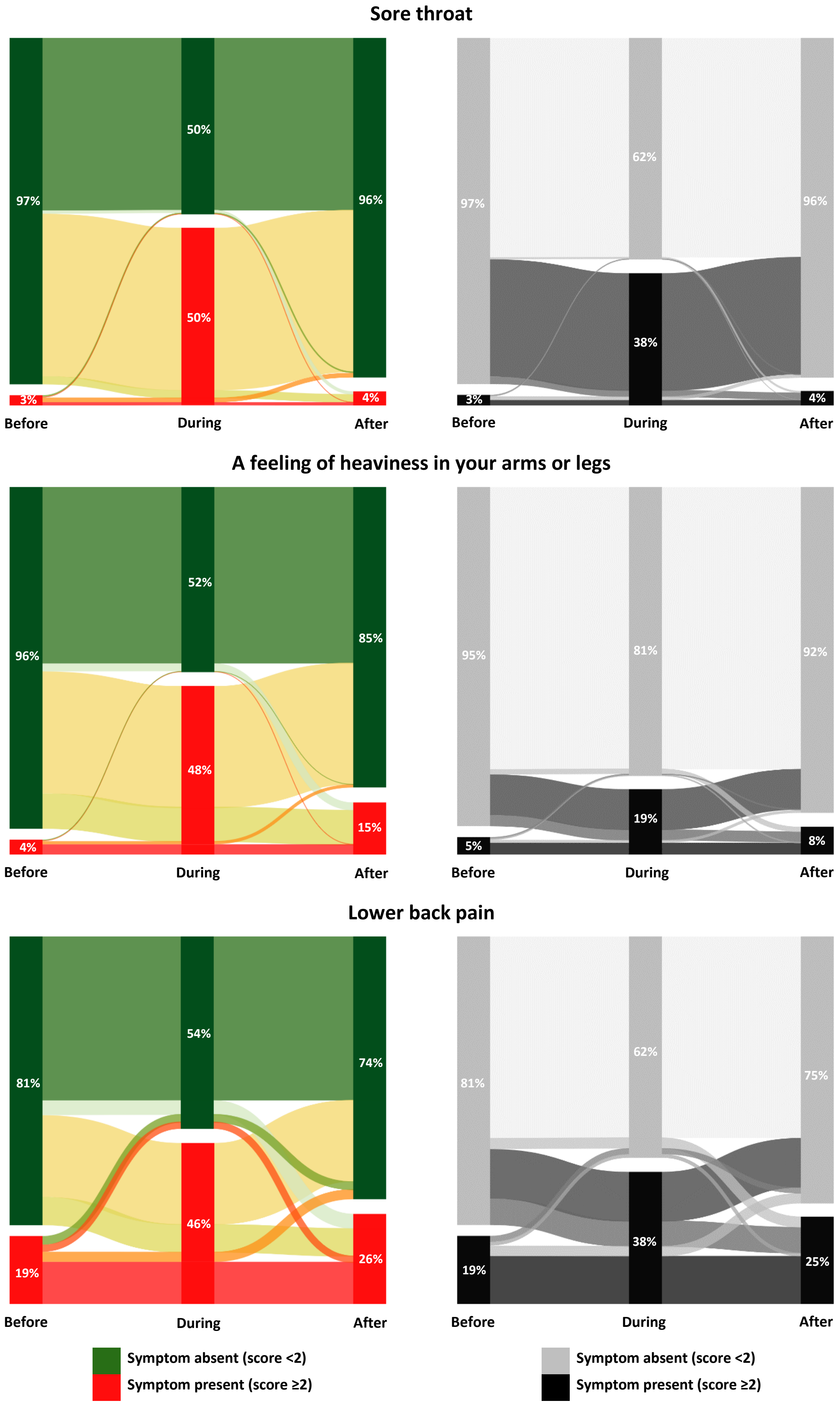
Sore throat
97%
3%
50%
50%
96%
4%
Before
During
After
97%
3%
62%
38%
96%
4%
Before
During
After
A feeling of heaviness in your arms or legs
96%
4%
52%
48%
85%
15%
Before
During
After
95%
5%
81%
19%
92%
8%
Before
During
After
Lower back pain
81%
19%
54%
46%
74%
26%
Before
During
After
81%
19%
62%
38%
75%
25%
Before
During
After
Symptom absent (score <2)
Symptom present (score ≥2)
Symptom absent (score <2)
Symptom present (score ≥2)

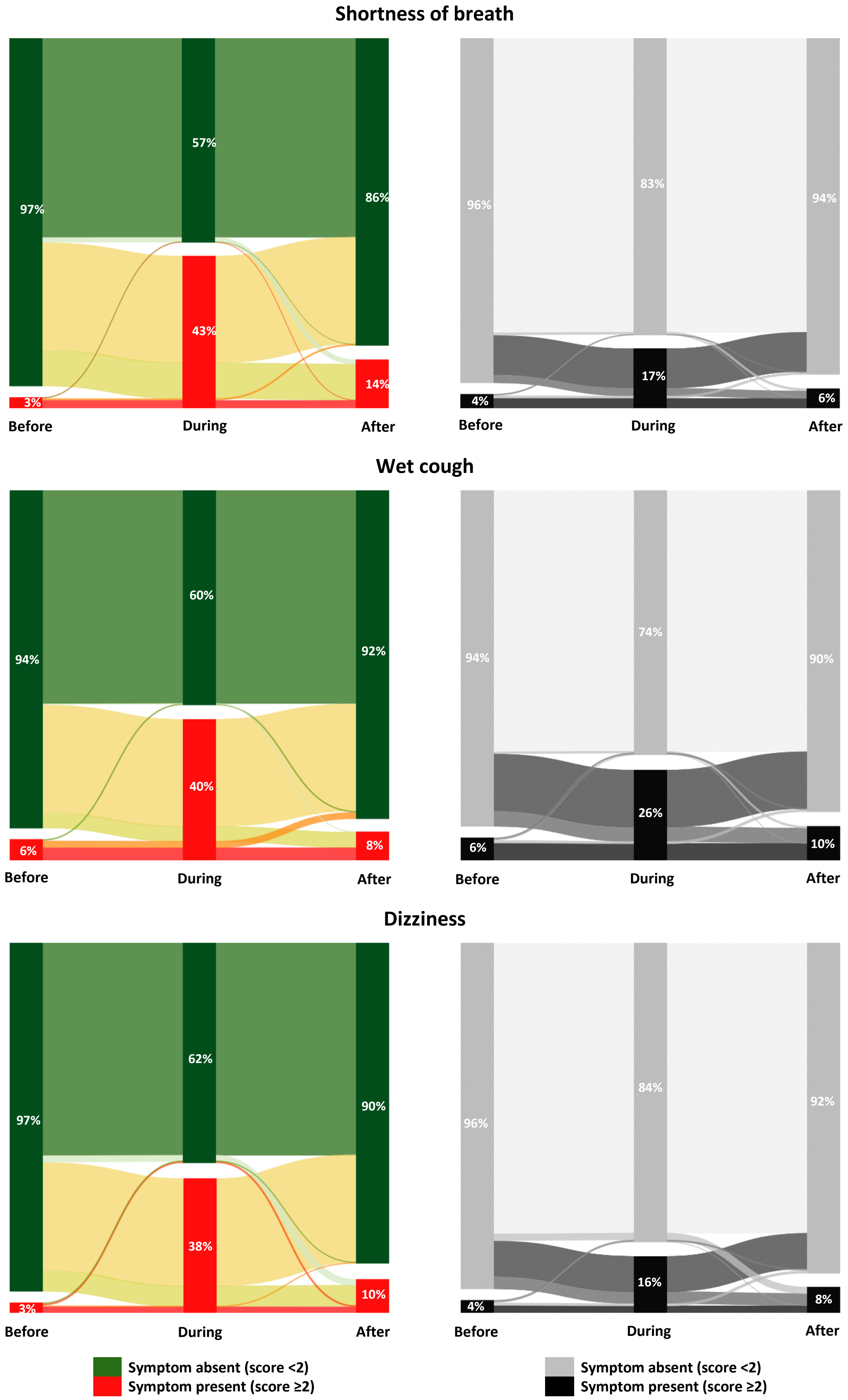

Shortness of breath
97%
3%
57%
43%
86%
14%
Before
During
After
96%
4%
83%
17%
94%
6%
Before
During
After
Wet cough
94%
6%
60%
40%
92%
8%
Before
During
After
94%
6%
74%
26%
90%
10%
Before
During
After
Dizziness
97%
3%
62%
38%
90%
10%
Before
During
After
96%
4%
84%
16%
92%
8%
Before
During
After
Symptom absent (score <2)
Symptom present (score ≥2)
Symptom absent (score <2)
Symptom present (score ≥2)

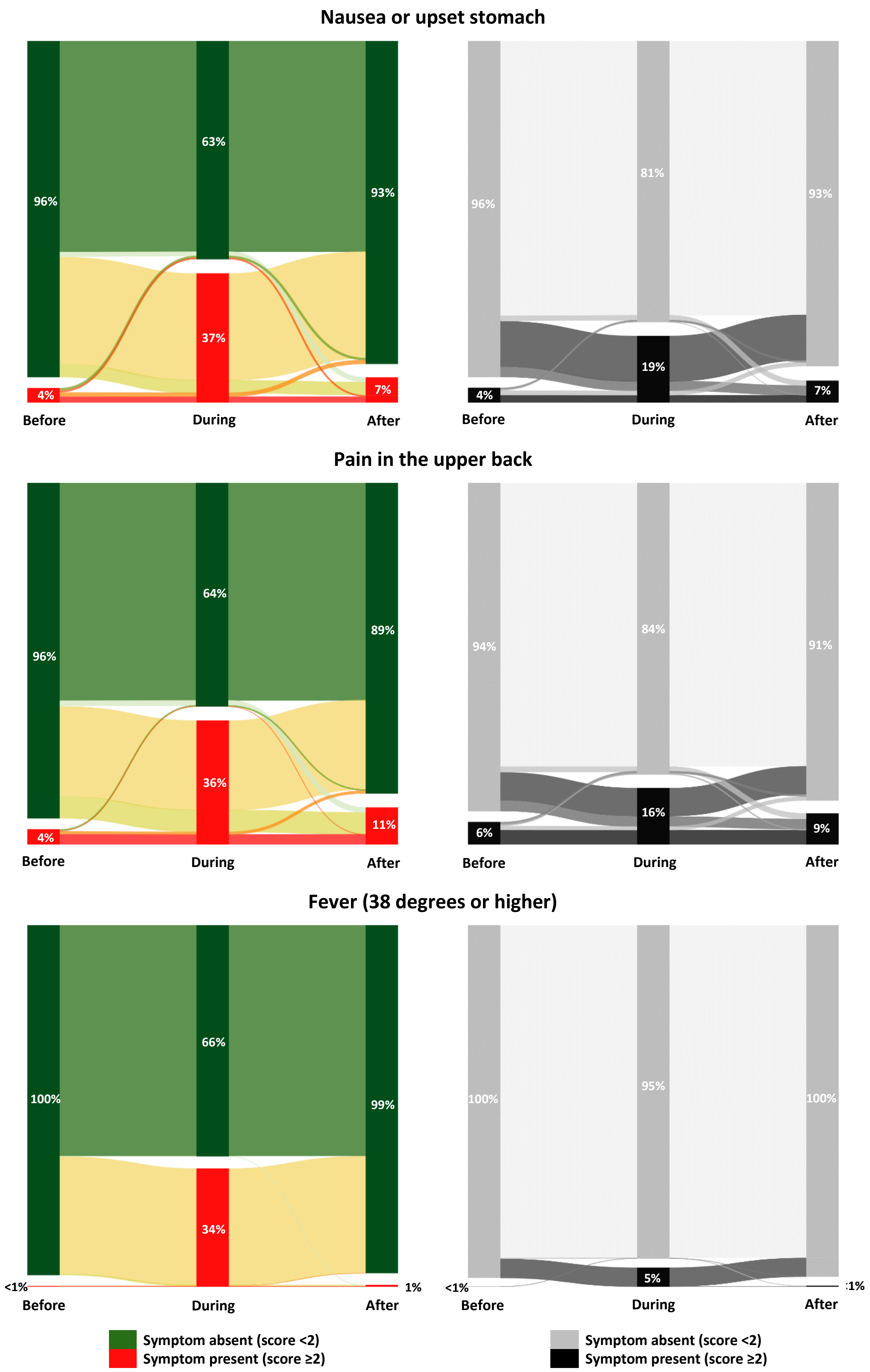

Nausea or upset stomach
96%
4%
63%
37%
93%
7%
Before
During
After
96%
4%
81%
19%
93%
7%
Before
During
After
Pain in the upper back
96%
4%
64%
36%
89%
11%
Before
During
After
94%
6%
84%
16%
91%
9%
Before
During
After
Fever (38 degrees or higher)
100%
<1%
66%
34%
99%
1%
Before
During
After
100%
<1%
95%
5%
100%
Before
During
After
Symptom absent (score <2)
Symptom present (score ≥2)
Symptom absent (score <2)
Symptom present (score ≥2)

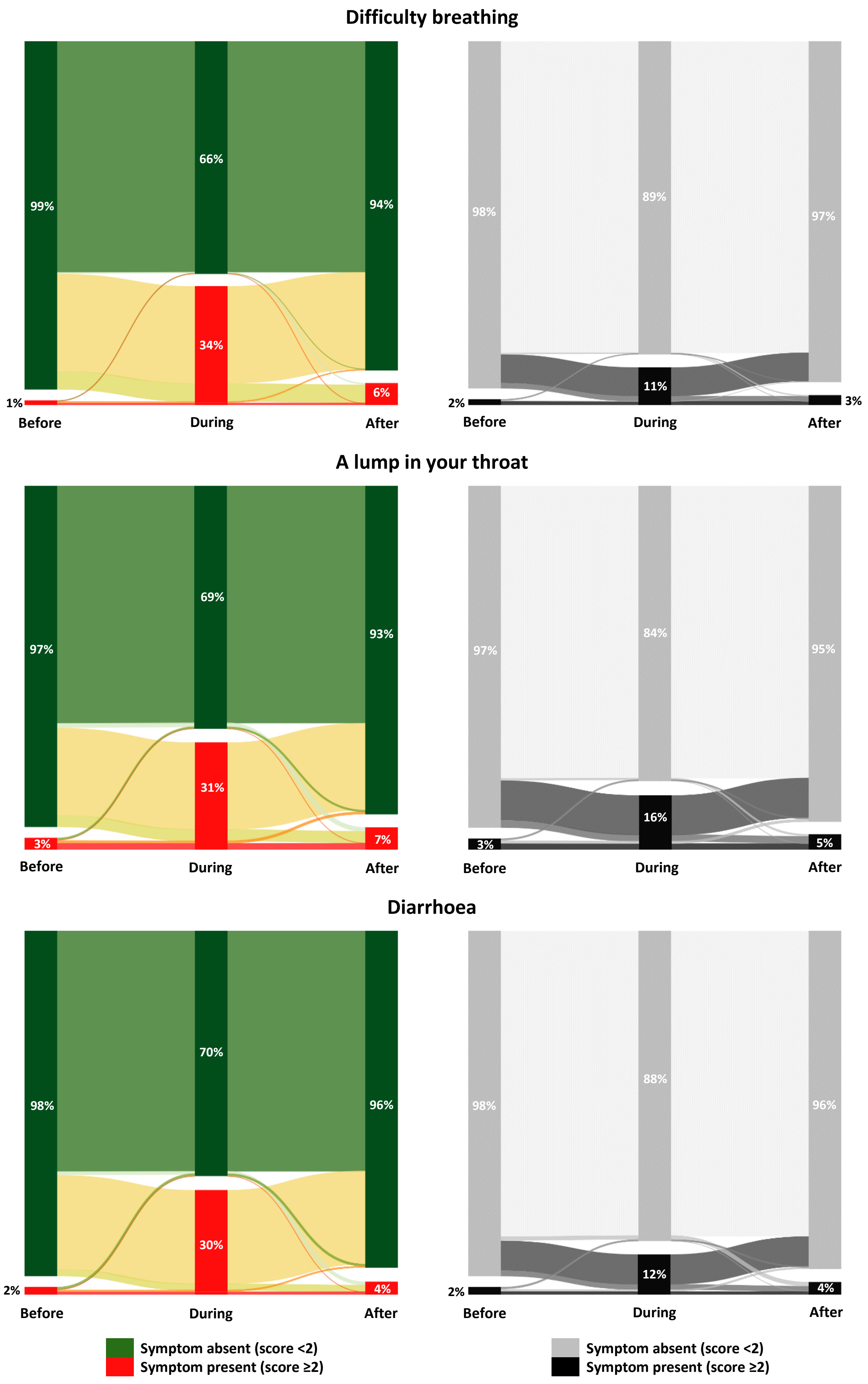

Difficulty breathing
99%
1%
66%
34%
94%
6%
Before
During
After
98%
2%
89%
11%
97%
3%
Before
During
After
A lump in your throat
97%
3%
69%
31%
93%
7%
Before
During
After
97%
3%
84%
16%
95%
5%
Before
During
After
Diarrhoea
98%
2%
70%
30%
96%
4%
Before
During
After
98%
2%
88%
12%
96%
4%
Before
During
After
Symptom absent (score <2)
Symptom present (score ≥2)
Symptom absent (score <2)
Symptom present (score ≥2)

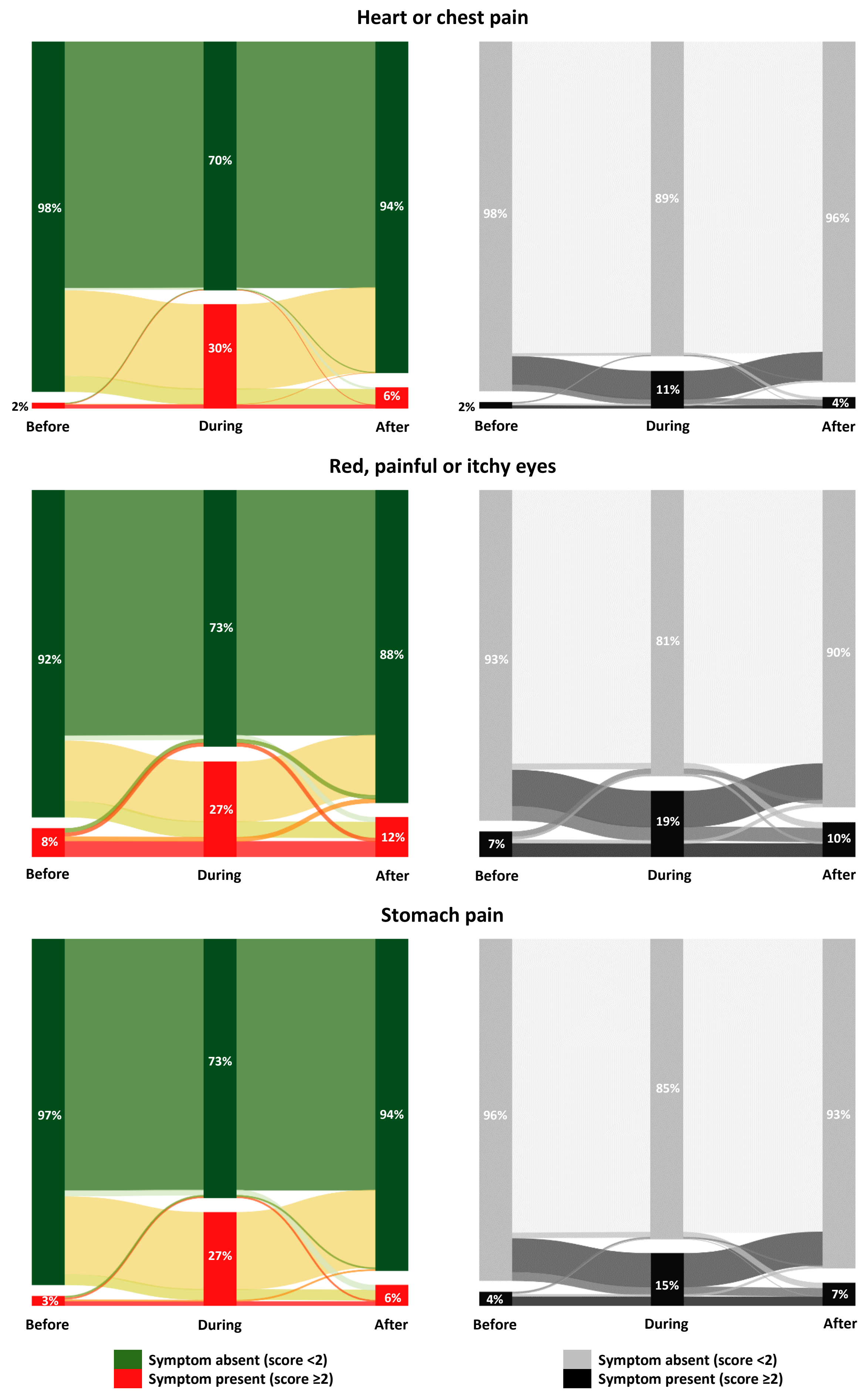
Heart or chest pain
98%
2%
70%
30%
94%
6%
Before
During
After
98%
2%
89%
11%
96%
4%
Before
During
After
Red, painful or itchy eyes
92%
8%
73%
27%
88%
12%
Before
During
After
93%
7%
81%
19%
90%
10%
Before
During
After
Stomach pain
97%
3%
73%
27%
94%
6%
Before
During
After
96%
4%
85%
15%
93%
7%
Before
During
After
Symptom absent (score <2)
Symptom present (score ≥2)
Symptom absent (score <2)
Symptom present (score ≥2)

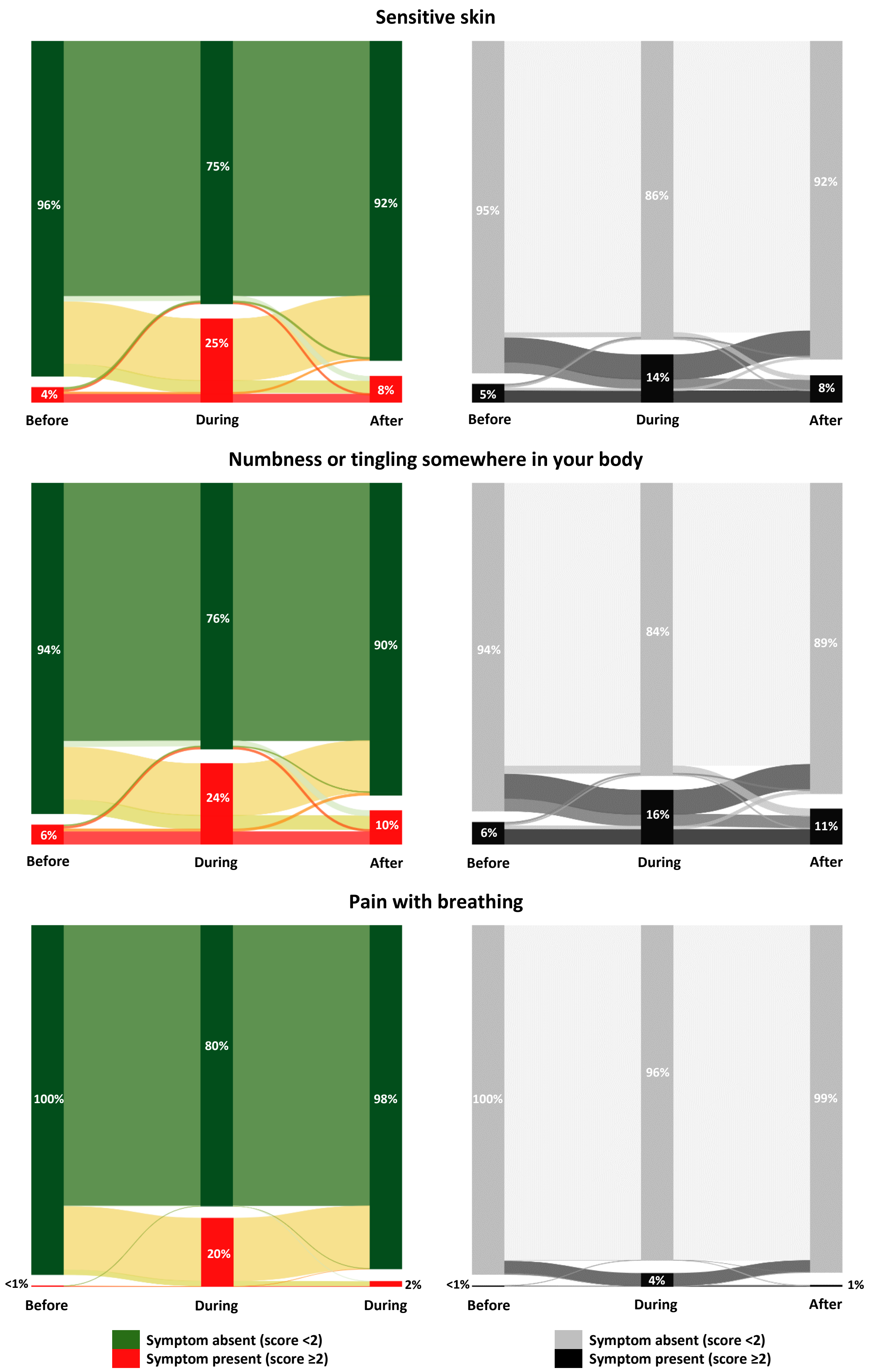
Sensitive skin
96%
75%
92%
25%
4%
8%
95%
86%
92%
14%
5%
8%
Before
During
After
Before
During
After
Numbness or tingling somewhere in your body
94%
76%
90%
24%
6%
10%
94%
84%
89%
16%
6%
11%
Before
During
After
Before
During
After
Pain with breathing
100%
80%
98%
20%
<1%
2%
100%
96%
99%
4%
<1%
1%
Before
During
During
Before
During
After
Symptom absent (score <2)
Symptom present (score ≥2)
Symptom absent (score <2)
Symptom present (score ≥2)

**Figure 2 Online Supplement: Prevalence and change of symptoms before, during, and after a SARS-CoV-2-PCR positive (left panels) and negative (right panels) test. The width of the lines is proportional to the flow rate.**
